# Impact of psychological stress on the outcomes of assisted reproduction in Tunisian infertile women

**DOI:** 10.11604/pamj.2021.40.250.32207

**Published:** 2021-12-21

**Authors:** Amira Sallem, Habiba Essoussi, Henda Ben Mustapha, Monia Zaouali, Mounir Ajina

**Affiliations:** 1 Laboratory of Histology Embryology and Cytogenetics (LR 40 ES 18), Faculty of Medicine, University of Monastir, Monastir, Tunisia,; 2 Laboratory of Cytogenetics and Reproductive Biology, Maternity and Neonatology Center, Fattouma Bourguiba University Teaching Hospital, Monastir, Tunisia,; 3 Department of Cytogenetic and Reproductive Biology, Farhat Hached University Hospital, Sousse, Tunisia,; 4 Unit of Reproductive Medicine, Farhat Hached University Teaching Hospital, Sousse, Tunisia,; 5 Laboratory of Physiology and Functional Exploration, Faculty of Medicine, Sousse, Tunisia

**Keywords:** Anxiety, beck anxiety inventory, cortisol, infertile women

## Abstract

**Introduction:**

childlessness is an emotionally difficult experience for infertile couples. Undergoing assisted reproductive treatments (ART) could generate further stress in these patients. Studies investigating the impact of anxiety on ART outcomes have shown controversial results. Moreover, there are no publications focusing on anxiety symptomsin infertile Tunisian couples.

**Methods:**

we conducted a prospective study including 79 infertile women undergoing in vitro fertilization at the Reproductive Medicine Unit of the Farhat Hached Hospital (Tunisia). Participants were asked to answer to the Beck anxiety inventory (BAI) on the day of oocyte retrieval. Accordingly, they were classified into the 3 groups: group A: very low anxiety (n= 36; BAI<21), group B: moderate anxiety (n= 24; 22≤BAI≤35) and group C: severe anxiety (n=19; BAI≥36). For each patient, two blood samples were collected to assess free cortisol level on the day of oocyte retrieval and on the day of embryo transfer.

**Results:**

results showed that women with primary infertility were significantly more stressed than those with secondary infertility (p= 0.011). Cortisol level was significantly higher on the day of embryo transfer than on the day of oocyte pick-up (p<0.0001). A lower implantation rate was found in severely anxious patients compared with moderately anxious women (p= 0.03) and those having low levels of anxiety (p= 0.001) and was negatively correlated to BAI score (r= -0.65; p= 0.001). Both clinical pregnancy and livebirth rates were similar among the three groups.

**Conclusion:**

the day of embryo transfer is the most stressful timepoint and psychological counseling is crucial to enhance implantation rate. Hence implantation took place, no effect of stress on pregnancy and live birth was found.

## Introduction

Infertility is a common health issue that affects 10-15% of couples. The powerlessness to conceive children is one of the most frustrating experiences in a couple life. Infertility has been found to be a stressor that can give rise to psychological difficulties such as lower self-esteem, feeling of isolation and even depression. In addition, the process of assisted reproduction technologies (ART) could result in greater stress and couples who try to conceive via ART are perceived to have high levels of anxiety [1,2]. Infertility is a stressful emotional experience for both male and female partners of infertile couples. However, recent reviews of the experiences of infertile couples in terms of gender differences have revealed that women had a more negative experience than men [3,4]. Earlier studies have examined the impact of anxiety on ART outcomes and the evidence for an association between anxiety and pregnancy rate after ART still to be weak [5].

Some researchers have also found an association between anxiety and ART outcomes [6,7] but not others [8]. Results of two meta-analysis including more than 30 studies revealed conflicting results on the topic [5,9]. In the developed countries, a particular attention has been paid to the psychological impact of infertility and thus the benefits of psychological counseling [10]. Unfortunately, little information is available on the psychological consequences of infertility in Tunisian couples and no published papers on the topic were found when screening literature data. Due to the above reasons, this study aimed to investigate the impact of anxiety on ART outcomes in Tunisian women undergoing In vitro fertilization (IVF)/Intracytoplasmic sperm injection (ICSI) cycles.

## Methods

**Study setting**: a prospective study.

**Study participants**: a total of 85 couples were initially recruited before the start of IVF/ICSI cycle at the Unit of Reproductive medicine of Farhat Hached University Teaching Hospital (Sousse, Tunisia). For all couples, ART procedure, the follow-up of the pregnancy and the delivery took place at our IVF unit. All patients who had an existing psychological problem were excluded. Were also excluded female partners with poor ovarian reserve (AMh<1 ng/ml), severe endometriosis and polycystic ovarian syndrome (SOPK). For the male partner, exclusion criteria were: akinetozoospermia, cryptozoospermia, monomorphic teratozoospermia as well as cycles with testicular sperm extraction. The study was approved by the clinical ethics committee of Farhat Hached University Teaching Hospital and all patients gave written informed consent to participate to the study. Prior to signing the consent form, patients were given detailed information about the study including mainly that participation is voluntary, and results are confidential as well as explanations of the study design and purpose.

**Psychological assessment**: state anxiety was measured by means of the valid French version of the Beck Anxiety Inventory (BAI) (21 anxiety symptoms) [11] which was self-administered forwomen just before oocyte retrieval. All items were scored on a scale ranging from 0 (I did not bother at all) to 3 (I almost could not stand it). The used questionnaire has shown satisfactory reliability and validity [12,13].

**Serum cortisol assessment**: blood samples were collected in a quiet room (between 8 and 9 a.m.) at two timepoints during the IVF cycle (T1: on the day of oocyte retrieval; just before oocyte retrieval and T2: on the day of embryo transfer; just before embryo transfer). After centrifugation, tubes were stored at -20°C until serum cortisol assessment by radioimmunoassay (RIA) at the Laboratory of physiology of The Faculty of Medicine of Sousse, Tunisia.

**Hormones assessment**: on day 3 of the menstrual cycle, hormonal analyses were performed including FSH, LH, E2, PRL according to standard protocols.

**IVF treatment**: programmed superovulation protocol has been performed with gonadotrophin-releasing hormone (GnRH) agonist (short or long) or antagonist and ovarian stimulation with follicle stimulating hormone (FSH) (Gonal-F^®^; Merck Serono) by subcutaneous injection. Monitoring was carried out by transvaginal sonography. When adequate stimulation was achieved (≥3 follicles of ≥18 mm in diameter), oocyte retrieval has been performed 34-36 h after administration of recombinant human chorionic gonadotropin. According to epidemiological factors (ages of partners, infertility duration and origin) as well as the semen status, conventional IVF or ICSI was performed. Embryo quality was evaluated on day 2 post-fertilization and the best embryo was transferred either on day 2 or on day 5 in case of extended embryo culture. A positive ß-hCG 14days after embryo transfer was diagnosed as a biochemical pregnancy and clinical pregnancy was defined as the observation of gestation sac with fetal echoes and pulsations on transvaginal sonography 7 weeks after embryo transfer.

**Statistical analysis**: statistics were compiled using Statistical package for Social Sciences, version 20.0 (SPSS 20.0). Comparisons between the three groups according to the BAI score were performed using Anova test. The Pearson method was used to evaluate the relationship between anxiety (as assessed by cortisol level and BAI) and ART outcomes. A p-value ≤0.05 was considered as statistically significant.

**Ethics approval**: the study was approved by the clinical ethics committee of Farhat Hached University Teaching Hospital.

## Results

**Figure 1:**
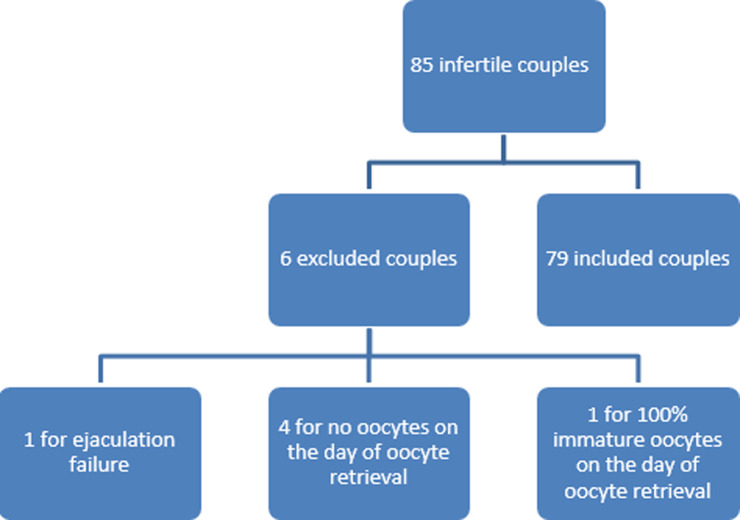
flow chart for couples’ inclusion process

Among the 85 couples initially included in the current study, 6 were excluded on the day of oocyte retrieval (1 patient with 100% of immature oocytes, 4 patients with no oocytes and 1 patient for ejaculation failure). The process of cohort selection is detailed in Figure 1. The remaining 79 couples were divided into three groups according to the BAI score of the female partner: group A: very low anxiety (n= 36; BAI≤21), group B: moderateanxiety (n= 24; 22≥BAI≤35) and group C: severe anxiety (n=19; BAI≥ 36).

**Patient´s characteristics and hormonal analysis**: the three groups were statistically comparable with regards to the two partners mean ages. The body mass index (BMI) of female partner was similar between groups and none of the included women was smoker. The distribution of fertility origin (male, female, both or idiopathic) in the three compared groups was similar. All patients belonging to group C (the most stressed ones) had a history of primary infertility contrary to those who have very low anxiety level (group A) (p= 0.011). Interestingly, women having the shortest infertility duration were severely anxious (group C) and the delay for that group was significantly different from that of group A women (p= 0.032). Day 3 hormonal analysis results for the female partner (including AMH, FSH, LH, estradiol (E2) and prolactin (PRL) levels) were similar between groups except the mean FSH level that was significantly elevated in case of moderate anxiety in comparison with severely anxious patients. These data are detailed in Table 1.

**Table 1:** comparison of demographic and laboratory features of study population among the three groups divided according to BAI scores

		Groups			Intergroup comparisons: P-value		
		Group A (n= 36)	Group B (n= 24)	Group C (n= 19)	A vs B	A vs C	B vs C
**Age (years)**	Women	35.27±5.27	33.86±5.84	34.36±5.07	0.346	0.541	0.771
	Men	41.25±5.93	40.09±7.96	38.78±5.18	0.530	0.133	0.546
**Women BMI**		28.62±3.13	28.86±5.95	30.00±5.02	0.875	0.262	0.548
**Day 3 hormonal assessment for female partners**	AMH(ng/ml)	3.18±2.97	3.25±2.88	2.76±2.46	0.933	0.627	0.592
	FSH (UI/l)	8.05±3.30	9.45±3.83	7.15±2.53	0.154	0.309	0.032*
	LH (UI/l)	3.81±2.12	6.27±4.04	4.73±2.70	0.019	0.200	0.191
	E2 (pg/ml)	35.9±18.10	32.94±11.33	28.66±11.08	0.525	0.101	0.182
	PRL (ng/ml)	23.33±12.56	37.61±57.71	19.5±7.94	0.265	0.184	0.160
**Infertility origin**	Male(%)	27.78%(n=10)	37.5%(n=9)	26.32%(n=5)	0.61	0.29	0.61
	Female (%)	36.11%(n=13)	41.66%(n=10)	52.64%(n=10)			
	Both (%)	25%(n=9)	12.5%(n=3)	5.26%(n=1)			
	Idiopathic (%)	11.11%(n=4)	8.34%(n=2)	15.78%(n=3)			
**Infertility duration (years)**		6.75±4.19	6.5±3.51	4.68±2.58	0.813	0.032*	0.071
**Infertility type**	Primary	72.2%(n=26)	87.5%(n=21)	100%(n=19)	0.15	0.011*	0.41
	Secondary	27.8%(n=10)	12.5%(n=3)	0%(n=0)			

* : Statistically significant difference (p<0.05), Data are presented as means ± SD or frequencies (%)

**Anxiety evaluation**: the mean BAI score of all included patients was of 22.35±14.17 (min= 0; max= 45). The mean cortisol serum level of the total population was significantly higher on the day of embryo transfer than that measured on the day of oocyte pick-up (139.24±50.45µg/dl vs 100.27±41.25 µg/dl respectively; p<0.0001). Embryo transfer was also more stressful than oocyte retrieval when comparing cortisol level at these two timepoints separately in each of the three groups (Table 2). Intergroup comparison revealed that on the day of embryo transfer, cortisol level was the most elevated in severely anxious women compared to the other two groups and the difference was statistically significant when patients of group C were compared to those of group A (the less stressed ones) with a p-value= 0.002. Interestingly, when considering the 79 recruited couples, anxiety as evaluated by the BAI was positively correlated with serum cortisol level on the day of embryo transfer (r= 0.29; p=0.008). Details of serum cortisol level at the two considered timepoints are shown in Table 3.

**Table 2:** comparison of mean cortisol level between the day of oocyte retrieval (T1) and the day of embryo transfer (T2) in each of the three groups

	T1 Cortisol level (µg/dl)	T2 Cortisol level (µg/dl)	Intragroup comparisons: P-value
Group A	95.47±31.97	121.80±34.58	0.001*
Group B	103.00±41.75	144.37±60.33	0.001*
Group C	105.94±55.40	165.78±51.67	0.001*

T1: cortisolemia: on the day of oocyte retrieval; T2: cortisolemia: on the day of embryo transfer, *: Statistically significant difference (p<0.05), Data are presented as mean ± SD

**Table 3:** comparison of mean cortisol level on the day of oocyte retrieval (T1) and on the day of embryo transfer (T2) among the three groups divided according to BAI scores

	Groups			Intergroup comparisons: P-value		
	Group A(n= 36)	Group B(n= 24)	Group C(n= 19)	A vs B	A vs C	B vs C
T1 Cortisol level (µg/dl)	95.47±31.97	103.00±41.75	105.94±55.40	0.433	0.454	0.843
T2 Cortisol level (µg/dl)	121.80±34.58	144.37±60.33	165.78±51.67	0.106	0.002*	0.226

T1 cortisolemia: on the day of oocyte retrieval, T2 cortisolemia: on the day of embryo transfer; *: Statistically significant difference (p<0.05); Data are presented as mean ± SD

**ART procedures and controlled ovarian stimulation details**: the three groups were also compared with regards to the type of ART procedure (conventional IVF, ICSI or both) as well as ovarian stimulation details. The only two significant differences were found between group A and C (p= 0.014) and between group B and C (p= 0.009) with regards to the distribution of the used ovarian stimulation protocols. The mainly used protocol was the short agonist protocol in group A and B and GnRH antagonist protocol in group C. The three groups were comparable when considering endometrial thickness and estradiol level on the day of ovulation triggering. Performed ART procedures as well as ovarian stimulation details are shown in Table 4.

**Table 4:** ART procedures and ovarian stimulation details among the three compared groups divided according to BAI scores

		Groups			Intergroup comparisons: P-value		
		Group A (n= 36)	Group B (n= 24)	Group C (n= 19)	A vs B	A vs C	B vs C
**ART**	cIVF	30.55%(n=11)	25%(n=6)	10.52%(n=2)	0.61	0.24	0.27
**Procedure**	ICSI	66.67%(n=24)	75%(n=18)	84.22%(n=16)			
	Half-ICSI	2.78%(n=1)	0%(n=0)	5.26%(n=1)			
**Ovarian stimulation protocol**	Antagonist	36.11%(n=13)	29.16%(n=7)	73.68%(n=14)	0.37	0.014*	0.009*
	Short agonist	38.88%(n=14)	58.33%(n=14)	10.53%(n=2)			
	Long agonist	22.22%(n=8)	8.33%(n=2)	5.26%(n=1)			
	Mild stimulation	2.79%(n=1)	7.18%(n=1)	10.53%(n=2)			
**Endometrial thickness (mm)**		9.80±2.49	9.31±1.80	9.52±1.60	0.446	0.667	0.708
**E2 on the day of ovulation triggering (UI/l)**		1413.81±853.44	1651.77±1043.31	1236.31± 874.10	0.362	0.480	0.179

* : Statistically significant difference (p<0.05); Data are presented as mean ± SD or frequency (%)

**Conventional IVF and ICSI outcomes among the three groups according to the BAI score**: when comparing IVF and ICSI procedures for the 79 included couples, we observed that the mean number of retrieved oocytes was similar between the three groups: 4.78±3.28 in group A, 5.45±3.2 in group B and 4.58±3.43 in group C (A vs B: p= 0.44; A vs C: p= 0.83 and Bvs C: p= 0.4). Oocyte maturation rate decreased when the level of anxiety was higher but differences between groups didn´t reach statistical significance: 73.93±27.07% in group A, 66.79±32% in group B and 63.66±38.81% in group C (A vs B: p= 0.39; A vs C: p= 0.49; B vs C: p= 0.92). Both fertilization and segmentation rates were similar among the three groups. The lowest men value of cleavage stage obtained embryos as well as the percentage of TOP quality cleavage embryos were registered in the group of severely anxious women but differences between groups with regards to these two parameters didn´t reach the statistically significant values.

There was no embryo transfer for a total of 4 patients: one from group A and one from group C for fertilization failure; one patient from group B and another from group C because of early embryo development arrest. Hence, the percentage of cycles with embryo transfer was similar between the three groups. Both the mean number of transferred embryos and the quality of embryo transfer (easy/difficult embryo transfer) were also comparable between groups. Interestingly, the lowest implantation rate was observed in women with severe anxiety (group C) and was of 7.14% compared with 18.91% in group B and 21.56% in women with very low anxiety (group A). The difference was statistically significant between both group A and C (p= 0.001) and group B and C (p= 0.03). There was a tendency to reach statistical significance when comparing biochemical pregnancy rate per cycle between group A (33.33%) and group C (10.52%) with a p-value of 0.06. Although patients of group C had the lowest clinical pregnancy rate (11.76%) and the lowest live birth rate (11.76%) compared to the two other groups, the current study didn´t show any statistically significant difference between the compared groups with regards to these two parameters. ART outcomes among the three groups are detailed in Table 5.

**Table 5:** ART outcomes among the three compared groups divided according to BAI scores

		Groups			Intergroup comparisons: P-value		
		Group A (n= 36)	Group B (n= 24)	Group C(n= 19)	A vs B	A vs C	B vs C
Mean retrieved oocytes		4.78± 3.28	5.45± 3.20	4.58±3.43	0.446	0.83	0.404
Oocyte maturation rate (%)		73.93±27.07	66.79 ± 32.00	63.66±38.81	0.398	0.499	0.920
Fertilization rate (%)		72.37±34.02	65.36±29.99	70.90±32.58	0.290	0.712	0.616
Segmentation rate (%)		99.33±3.65	95.17± 16.03	98.71±4.62	0.288	0.845	0.880
Mean number of day2-3 obtained embryos (n)		2.49±2.30	2.29± 1.64	2.05± 2.09	0.870	0.484	0.469
Cleavage stage TOP embryo rate (%)		64.38±38.35	76.73±30.07	54.39± 34.27	0.236	0.420	0.061
Mean number of transferred embryos (n)		1.33±0.67	1.50±0.65	1.37±0.89	0.443	0.788	0.740
Cycles with embryo transfer		97.22% (n=35)	95.83% (n= 23)	89.47% (n= 17)	0.42	0.23	0.11
Embryo transfer quality	Easy	94.28% (n= 33)	78.26% (n= 18)	94.11% (n= 16)	0.18	0.48	0.27
	Difficult	5.71%(n= 2)	21.74%(n= 5)	5.89% (n= 1)			
Implantation rate(%)		21.56	18.91	7.14	0.26	0.001*	0.03*
Biochemical pregnancy per cycle (%)		33.33	25	10.52	0.49	0.06	0.23
Biochemical pregnancy per embryo transfer (%)		34.28	26.08	11.76	0.77	0.11	0.11
Clinical pregnancy per embryo transfer (%)		22.85	17.39	11.76	0.6	0.29	0.57
Live birth rate (%)		22.85	17.39	11.76	0.6	0.29	0.57

* : Statistically significant difference (p<0.05), Data are presented as means±SD or frequencies (%)

**Correlation study between anxiety and ART outcomes**: we have observed a negative significant correlation between BAI score and embryo segmentation (r= -0.24; p= 0.05) as well as BAI score and implantation rate (r= -0.65; p= 0.001). No correlations were found between serum cortisol level on the day of oocyte retrieval and ART outcomes in our cohort of patients. However, when measuring cortisol concentration on the day of embryo transfer, we noticed a negative correlation between this latter and embryo segmentation rate (r= -0.29; p= 0.019). No further correlations were found. Table 6 summarizes correlation study between BAI as well as T1 and T2 cortisolemia and ART outcomes.

**Table 6:** correlation study between BAI, T1 and T2 cortisolemia and ART outcomes in the 79 followed couples

	BAI score		T1 cortisolemia		T2 cortisolemia	
	r	p	r	p	r	p
Number of retrieved oocytes	–0.06	0.59	0.13	0.23	–0.12	0.28
Oocyte maturation rate	–0.13	0.25	0.09	0.44	–0.21	0.06
Fertilization rate	–0.19	0.11	–0.15	0.2	0.04	0.73
Segmentation rate	–0.24	0.05	0.08	0.52	–0.29	0.019
Number of obtained day 2-3 embryos	–0.1	0.4	0.07	0.53	–0.2	0.1
Number of day 2-3 Top quality embryos	–0.11	0.32	0.01	0.91	0.11	0.33
Number of transferred embryos	–0.02	0.85	0.07	0.48	–0.05	0.65
Implantation rate	–0.65	0.001	0.01	0.95	–0.35	0.09

T1: cortisolemia: on the day of oocyte retrieval; T2: cortisolemia: on the day of embryo transfer; Are shown in bold statistically significant correlations

## Discussion

Our data mainly showed that based on cortisol level assessment in Tunisian infertile women, the day of embryo transfer is more stressful than the day of oocyte retrieval and that severely anxious women have a significantly decreased implantation rate. Being involuntarily childless and going through various ART procedures imposes considerable stress on the couple [14] and especially for the female partner because literature data have shown that the picture seems to be gender-related [15,16]. This is a widely shared opinion [17,18]. As the female partner undergoes more stress during fertility management, we aimed in the current study to have an overview on women anxiety level starting from the day of oocyte retrieval to the day of embryo transfer and detailing ART outcomes.

When focusing on epidemiological data, our results showed that women with primary infertility were exposed to significantly higher level of stress than those with secondary infertility. All patients belonging to group C have a history of primary infertility. Those with secondary infertility seem to be less anxious probably because they have children at home to care for which may be one of infertility-coping strategies. Moreover, severely anxious women had the lowest infertility duration compared to those with very low anxiety (p= 0.032) which highlight the impact of anxiety on shortening the delay of infertility management and engagement in an ART treatment course. As we evaluated stress in our cohort of patients with two different tools (BAI score and serum cortisol level), it was of great interest to highlight the positive correlation between the two tests on the day of embryo transfer (r= 0.29; p= 0.008).

Our results have shown that infertile women were much more anxious on the day of embryo transfer, and this was confirmed in the three population groups. Moreover, the existence of a positive correlation between BAI results and serum cortisol concentration on the day of embryo transfer (r= 0.29; p=0.008) provides a supplemental evidence in relation with the intensity of perceived anxiety on that precise moment during an IVF procedure. This is in line with data published by Lin and collaborators [19] who have shown that women experienced more somatic symptoms (abdominal distention, breast engorgement, nausea, faintness, diarrhea, sleep disturbance) and psychological distress on the day of embryo transfer. However, other reports considered the day of oocyte retrieval and pregnancy test to be the most stressful stages of an IVF cycle [20,21]. Embryo transfer is a crucial step in IVF cycle and whether the transferred embryo will implant or not is a determinant element in the ART procedure outcomes. It would be of great interest to evaluate anxiety for both partners on the day of pregnancy test which was not assessed in the current study. Although the results concerning the most stressful moment during an IVF cycle have been mixed, authors have generally reached the conclusion that infertility is associated with periodically heightened levels of psychological symptoms of distress, depression and anxiety [22]. It´s particularly important for the medical staff managing infertility issues to be aware that infertile patients express distinct need for emotional support and so require individualized psychological care. This is in relation to their medical history, sociodemographic and behavioral characteristics, personality trait, adaptability, cultural expectations, and social support systems. Hence, the fertility staff must provide patients with understandable and customizedexplanations about treatment results and treatment options [23,24]. Nowadays, the covid-19 pandemic context was shown to generated higher distress levels in infertile couples [25] who need specific psychological support.

It has been reported that some infertility patients suppress their feelings of stress because they want to show the clinic that they are functioning well both socially and psychologically [26]. So, it is important to diagnose patients who fail to cope with infertility and ART. The ESHRE guideline also recommends referring patients at risk of emotional problems to specialized psychosocial care (infertility counselling or psychotherapy)before the start of IVF/ICSI treatment [10]. According to the results of our study we suggest providing infertile women with psychological support before embryo transfer. One of the main questions that we aimed to answer through the current study was: to which extent may anxiety negatively affect ART outcomes? There is considerable debate concerning the impact of anxiety on ART outcomes. Unfortunately, in many developing countries such as Tunisia this question is not yet openly asked and so, little information is available on the consequences of emotional stress on ART outcomes. Apart from the study of El Kissi et collaborators which compared in 2013 the level of anxiety perceived by Tunisian infertile men and women without analyzing the impact of the observed stress on ART results [27], this is to the best of our knowledge, the first study carried out in Tunisia to understand the possible links between these two parameters by two different tools.

When focusing on ART outcomes, the decrease in implantation rate between patients having very low and moderate anxiety was not statistically significant (p= 0.26). However, the decrease of that same parameter was statistically significant when women were exposed to high levels of anxiety (from 21.56% in group A to 7.14% in group C; p= 0.001 and from 18.91 in group B to 7.14% in group C; p= 0.03). Furthermore, an important finding of the current study is that psychological stress as evaluated by BAI was negatively correlated to implantation rate (r= -0.65; p= 0.001) which highlighted the negative impact of anxiety on embryo implantation. Implantation process is very complex at the molecular level and involves multiple factors in relation with embryo quality, endometrial receptivity and the interaction between these two entities. Our results are in line with those of [4] who recently demonstrated a negative impact of female negative life events on embryo implantation. This could be explained by the effect of psychological stress on dysregulating the uterus microenvironment through promoting oxidative stress and inflammation which could alter endometrial receptivity and potentially lead to unsuccessful embryo implantation. Women suffering from major depression were shown to have a dysregulation of immune mediators such as rise of pro-inflammatory cytokines IL-1ß, IL-6 and decrease in anti-inflammatory cytokines such as TGF-ß [28]. A significant increase in serum IL-1ß leading to an inhibition of progesterone production by luteal cells is thought to be responsible for blastocyst attachment inhibition in women with recurrent implantation failure in IVF [4,29,30].Serum TGF-1ß level was found to be negatively correlated with female stress and depression. As TGF-ß is involved in progesterone synthesis regulation and trophoblast invasion and proliferation, stress related decrease in serum TGF-ß concentration may be one of the reasons leading to implantation failure in anxious women [4]. These data support the immune-endocrine theory associating stress and impaired fertility.

Once the implantation stage is passed, and apart from a tendency to significant decrease of biochemical pregnancy per cycle in women with severe anxiety (p= 0.06), we did not observe any statistically significant impact of stress on both clinical pregnancy and live-birth rates. These reassuring findings are in line with those published by [31] in 2018 on a large cohort of 485 Swedish women receiving fertility treatment. It has been established that infertility-related stress is mainly due to a suppression of the hypothalamic-puituitary-gonadal axis activity through an inhibition of gonadotropin-releasing hormone (GnRH) activity which results in a decrease in gonadotropin levels. Hence, anovulation is thought to be the mechanism by which stress leads to conception difficulties and IVF treatment is the way to overcome the issue of anovulation through ovarian stimulation [32]. This hypothesis could explain the absence of correlation between stress and both pregnancy and live-birth rates in our study. The only two studies that have concluded to a link between serum cortisol and clinical pregnancy following an IVF cycle are those of Demyttenaere in 1992 [33] and An in 2013 [34]. A meta-analysis conducted by Frederiksen *et al*. [14] had established that women receiving some form of psychological intervention when treated for infertility are approximately twice as likely to become pregnant when compared with controls. Authors had concluded that psychological interventions were effective in reducing emotional distress as well as increasing the conception rate [35,36]. In the same context, various complementary practices based on relaxation techniques aiming to increase blood circulation and stimulating the energy in the reproductive organs and the pelvic area such as yoga were efficient in improving ART outcomes [37-39].

The main limitation of the current study was that it didn´t record women´s education level and socioeconomic profile to see whether these parameters could interfere with infertility-related stress in Tunisian population. Meanwhile, it has the advantage of including a homogenous population with no factors known to affect only IVF outcomes such as obesity or both IVF outcomes and cortisol level such as smoking. In the design of our study, patients with polycystic ovary syndrome (PCOS) were discarded as they are more likely to experience high distress level mainly due to obesity and lack of sexual satisfaction when coping with infertility [16]. When dividing the three groups based on BAI score, patients were comparable with regards to demographic features.

## Conclusion

Undeniably, psychological counseling is crucial for couples seeking infertility treatment. The day of embryo transfer was defined as a real moment of stress for infertile women. Psychological care before embryo transfer could help to enhance the shown decrease in implantation rate. Hence implantation has occurred, no obvious impact of stress on pregnancy and live birth was seen.

### What is known about this topic

- *Controversies with regards to the link between anxiety and ART outcomes;*
- *The only available study (published on 2013) on the impact of anxiety on infertile Tunisian patients has shown that women endorsed higher psychological distress when compared to men*.

### What this study adds

- *This is to the best of our knowledge, the first study carried out in Tunisia to investigate the impact of anxiety on ART outcomes;*
- *The day of embryo transfer is more stressful than the day of oocyte retrieval;*
- *Female partner anxiety negatively impacts implantation rate without significant impact on pregnancy and live-birth rates*.
